# *Cassia auriculata*: Aspects of Safety Pharmacology and Drug Interaction

**DOI:** 10.1093/ecam/nep237

**Published:** 2011-05-03

**Authors:** Amrutesh S. Puranik, Ganesh Halade, Sandeep Kumar, Ranjan Mogre, Kishori Apte, Ashok D. B. Vaidya, Bhushan Patwardhan

**Affiliations:** ^1^Interdisciplinary School of Health Sciences, University of Pune, Pune 411007, India; ^2^University of Texas Health Science Center at San Antonio, San Antonio, TX, USA; ^3^National Center for Cell Sciences, Pune, India; ^4^Applied Biotech, Vile Parle, Mumbai, India; ^5^National Toxicology Center, Pune, India; ^6^ICMR—Advanced Centre of Reverse Pharmacology, MRC-KHS, Vile Parle, Mumbai, India; ^7^Manipal Education, Bangalore 560008, India

## Abstract

Safety pharmacology studies help in identifying preclinical adverse drug reactions. We carried out routine safety pharmacology with focus on cardiovascular variables and pharmacokinetic herb-drug interaction studies on rats fed with standardized traditional hydro-alcoholic extract and technology-based supercritical extract of *Cassia auriculata* for 12 weeks. Our studies indicate that both these extracts are pharmacologically safe and did not show any significant adverse reactions at the tested doses. The traditional hydro-alcoholic extract did not show any significant effect on pharmacokinetics; however, the technology-based supercritical extract caused a significant reduction in absorption of metformin. Our results indicate the need to include pharmacokinetic herb-drug interaction studies as evidence for safety especially for technology-based extracts.

## 1. Introduction

While the dietary botanical supplement market is growing, the need for more rigorous clinical and scientific research on herbal and traditional medicine is strongly advocated for larger acceptances and visibility [1]. Traditional herbal medicines have a long history of use and are generally considered to be safer than synthetic drugs. Traditional medicine-inspired approaches remain important especially for the management of chronic diseases as well as to facilitate natural product drug discovery [2, 3].

Combinations of herbal medicines or phytochemical actives are found to be beneficial in certain diseases when given along with modern synthetic drugs [4]. However, during concurrent use with modern medicines some potential adverse reactions have been reported [5, 6]. Herbal medicines when co-administered with synthetic drugs may result in herb-drug interactions influencing bioavailability leading to adverse events [7]. Therefore, studies related to safety pharmacology and pharmacokinetic herb-drug interactions are more important when concurrent use of herbal and modern medicine is on rise. For chronic diseases including diabetes and cardiovascular conditions where long-term treatment is needed, co-administration of herbal and modern medicines may pose higher risk of adverse events and hence sufficient evidence of safety is necessary [8, 9]. In such situations, safety pharmacology is useful to predict the adverse drug reactions [10]. Safety pharmacology deals with key aspects that are causal to unknown adverse events and aims at investigating potential undesirable pharmacodynamic effects on physiological functions in the therapeutic dose range. Increasing regulatory concerns in drug development have broadened the scope of safety to include general pharmacology, adverse drug reactions, cardiovascular pharmacology and pharmacokinetic herb-drug interactions [11] (Figure 2).

Ayurveda advocates several medicinal plants for treatment of diabetes. We selected *Cassia auriculata* (CA) Linn, family Caesalpiniaceae, also known as Tanner's Cassia or *Avartaki* in vernacular, which is used in Ayurveda in the treatment of diabetes [12]. The flower and leaf extract of CA is shown as antihyperglycemic in streptozotocin-induced experimental diabetes [13, 14]. Methanolic extracts of CA flowers have been demonstrated to inhibit *α*-glucosidase *in vivo* as well as *in vitro* [15]. Its aqueous extract is reported to prevent the lipid peroxidation in brain of diabetic rats [16]. Hyponidd, a formulation containing CA has been shown as antihyperglycemic and antioxidant [17]. Polyphenols are considered to be the active ingredients of CA [18]. Generally, hydro-alcoholic solvent is used to extract polyphenols from the crude herbal material. Traditional medicine practice also generally advocates use of hydro-alcoholic (HA) extracts of herbal materials. Yet, there is a growing practice to adopt technology-based supercritical fluid extracts (SFE) for higher yields of polyphenol [19–21] and are now widely used by the industry [22–24]. However, technology-based extracts such as SFE has no prior evidence of traditional use and hence we attempted a safety pharmacology study.

Diabetes is a difficult to manage disease and long-term treatment with careful monitoring is necessary. It is known that many a times patients tend to explore several alternatives concurrently with anti-hyperglycemic agents such as metformin and insulin-sensitizing agents like thiazolidinediones for treatment of diabetes [25]. The Food and Drug Administration has made cardiovascular safety mandatory for any new therapeutic agents intended in the management of diabetes [26]. Therefore, while studying safety pharmacology we focused on the cardiovascular system. We also studied the pharmacokinetic herb-drug interaction of CA extracts with metformin, which is the most preferred first line treatment of type 2 diabetes. This study is very relevant and important because metformin is classified as a hard drug, which does not get metabolized in the body and therefore increases possibility of herb-drug interactions.

## 2. Materials and Methods

### 2.1. Plant Source

CA was collected from biological reserves of Sangamner area, District Ahmednagar, Maharashtra, India. Botanical authentication was carried out by qualified person at Agharkar Research Institute, Pune, India, and a voucher sample has been deposited at its herbarium.

### 2.2. Chemicals

All the standards including (–) epicatechin, (–) epicatechin gallate, procyanidin B1, (–) catechin, phenformin hydrochloride and homocysteine were procured from Sigma-Aldrich, USA, and the High performance liquid chromatography (HPLC) solvents from Merck, India. Metformin was obtained from LGC, Promochem, India. Sodium carboxy methyl cellulose (NaCMC) (Merck, India) was used as vehicle for administering CA extracts. The kits for serum analysis of glucose, total cholesterol (TC), triglycerides (TG), serum glutamic pyruvic transaminase (ALT or SGPT), serum glutamic oxatoacetic transaminase (AST or SGOT), alkaline phosphatase (ALP), blood urea nitrogen (BUN), total proteins albumin and globulin were purchased from Merck Ecoline, Mumbai, India. The serum total bilirubin and creatinine were estimated using kits from Labkit, Spain. Creatinine kinase-MB (CKMB) and troponin I was estimated by kits from Teko diagnostics, CA, USA, and Monobind Inc., CA, USA, respectively.

### 2.3. Extracts

The seeds of CA were crushed into a coarse powder. The powder was then defatted by petroleum ether prior subjecting it to hydro-alcoholic maceration for 7 days. The collected solvent was evaporated on a rotavapor at 35°C and the extract with a yield of 15% was freeze dried. This hydro-alcoholic extract of CA (CA-HA) was used for present study. The technology-based supercritical fluid extract of CA (CA-SFE) was prepared at Vedic Supercriticals & Biotechnologies (I) Pvt Ltd, Pune, India. The extraction was carried by modifying the reported method [27]. Briefly, carbon dioxide in the supercritical state at a flow rate of 100 kg h^−1^ with 3% ethyl alcohol as modifier at a pressure of 300 bars at 45°C was used for 2.5 h. The yield obtained was 3.8%.

### 2.4. Standardization

HPLC was used for standardization. CA-HA and CA-SFE extracts were injected into a pH stable reverse phase C18 column (4.6 × 250 mm, Varian, Varian Inc.) pre-equilibrated with mobile phase solution containing mobile phase A water : methanol : formic acid (79.5 : 20 : 0.5) and B acetonitrile : formic acid (99.7 : 0.3) at a flow rate of 0.4 mL and a gradient described in Supplementary Figure 1. The chromatographic system used for all the analysis (Dionex, Germany) consisted of P-680 quaternary gradient pump, an ASI 100 autosampler, a universal chromatographic interface UCI-50 and diode array detector 340U or fluorescence detector RF-2000 integrated by Chromeleon Software 6.70. Standard curves of (–) epicatechin, (–) catechin and procyanidin B1 are plotted and the concentration of unknown is quantified by linear regression.

### 2.5. Animals

Wistar albino rats of both sexes, aged 6–8 weeks, weighing 150–220 g were randomly bred in well-controlled animal house facility of National Toxicology Centre, Pune, India. The animals were housed in standard conditions of temperature (22 ± 5°C) and humidity (55 ± 15%) and 12 h light-dark cycles. They were fed on conventional laboratory pelleted diet and water *ad libitum*. All the procedures were performed as per the guidelines of the Committee for the Purpose of Control and Supervision of Experiments on Animals (CPCSEA), Ministry of Animal Welfare Division, Government of India, New Delhi, and was approved by the Institutional Animal Ethics Committee of National Toxicology Center, Pune, India.

### 2.6. Safety Studies

Animals were divided into six groups (*n* = 12 per group; six males and six females). CA-SFE and CA-HA extracts were re-suspended in 0.5% vehicle and administered daily orally by gavages using feeding needle to all the six groups. The control group received equal volume of vehicle. CA-SFE was administered at three doses: 250, 500 and 1000 mg kg^−1^ and CA-HA at two dose: 500 and 1000 mg kg^−1^ for a period of 12 weeks. The experimental doses were decided based on the human dose in traditional practice [11].

### 2.7. Variables Monitored

#### 2.7.1. Body Weight

Body weight of all the groups treated with CA-SFE, CA-HA extracts and control were monitored weekly.

#### 2.7.2. Organ Weight

The animals were sacrificed at the termination of the study. The organs such as liver, muscles, lungs, spleen, kidney, heart, brain, adrenals, testis and ovaries were removed, blotted free of blood, weighed immediately on precision balance CA123 (Clontech, India) and stored in 4% paraformaldehyde for subsequent histological analysis. All the organs were embedded in paraffin, sectioned and stained with hematoxylin-eosin.

#### 2.7.3. Hematology and Biochemistry

After 12 h fasting, blood was collected retro-orbitally under anesthesia. Blood was immediately analyzed for white blood cells (WBC), red blood cells (RBC), hemoglobin (HB), hematocrit (HCT), mean corpuscular volume (MCV), mean cell hemoglobin (MCH), mean cell hemoglobin concentration (MCHC) and platelet count (PLT) by using an automated hematology analyzer (Sysmex-KX-21, Kobe, Japan).

The serum was separated from blood cells by centrifugation at 2000 g for 15 min at 4°C Superspin R-V/F_M_, Plasto Crafts, Mumbai, India. For glucose evaluation, blood was collected in fluoride bulb to avoid glycolysis on storage. The serum samples were stored at −20°C until analyzed. Serum was assayed for glucose, cholesterol, triglyceride, total bilirubin, ALT, AST, ALP, BUN, creatinine, total proteins, albumin and globulin by using calibrated auto-analyzer Hitachi-704, Boehringer Mannheim Diagnostics, Germany. Serum homocysteine was analyzed by HPLC using a fluorescence detector [28, 29]. The limit of detection of homocysteine was 500 nmol L^−1^.

#### 2.7.4. Cardiovascular Studies

After 12 weeks of dosing CA extracts, rats were anesthetized with urethane (1 g kg^−1^ body weight). The right carotid artery was cannulated with a microtip pressure transducer (SPR-671, Millar Instruments) connected to an electrostatic chart recorder. The transducer was advanced into the left ventricle for the evaluation of ventricular pressures (systolic and diastolic) and heart rate were monitored and recorded using Chart 5.5 (ADI Instruments, Australia). Rectal temperature was maintained at 36–38°C throughout the procedure [30]. Troponin, homocysteine and CKMB were identified as biochemical markers for heart damage.

### 2.8. Pharmacokinetic Herb-Drug Interaction

**Figure 1:**
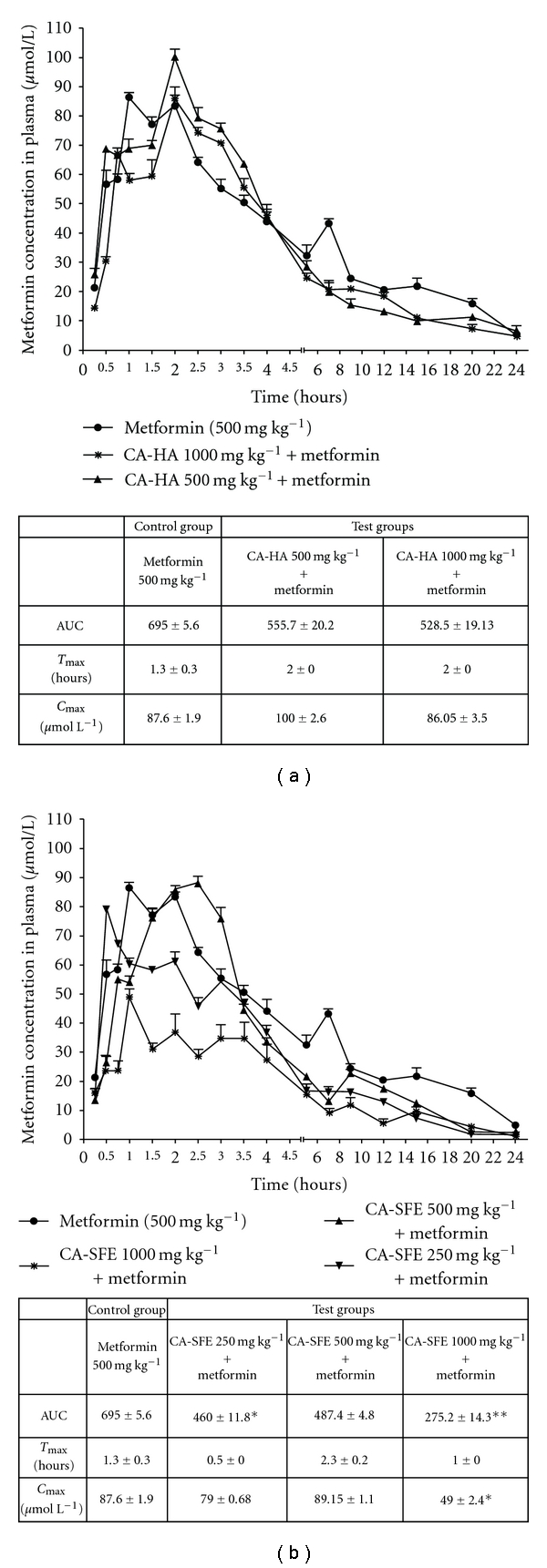
(a) Pharmacokinetic herb-drug interaction study on CA-HA extracts. Data are expressed as mean ± SE obrained from *n* = 4 rats per three time intervals. No significant reduction in AUC_M_ for CA-HA when compared with metformin group versus test group. (b) Pharmacokinetic herb-drug interaction study on CA-SFE extracts. Significant reduction in AUC_M_ for CA-SFE when compared with metformin group versus test group. Significance denoted by **P < 0.01, *P < 0.05.

In the control group, the pharmacokinetics of metformin at dose of 500 mg kg^−1^ was studied. In the test groups, CA-SFE at 250, 500 and 1000 mg kg^−1^ and CA-HA at 500 and 1000 mg kg^−1^ dose were orally co-administered with metformin (500 mg kg^−1^). Male wistar rats (*n* = 4 rats per three time interval, i.e., 28 rats per group) weighing 150–200 g were randomized. Blood (150 *μ*L) was collected at specified time (Figure 1) intervals over a period of 48 h. Plasma was separated from blood and stored at −30°C until metformin assay. The area under curve for metformin (AUC_M_) was calculated in all the groups. AUC_M_ in control group was compared with AUC_M_ in test groups. A decrease in AUC_M_ test versus AUC_M_ control was interpreted as the herb-drug interaction. Metformin was estimated using previously reported methods with phenformin as internal standard [31]. The limit of detection for metformin in plasma was 50 ng mL^−1^.

### 2.9. Statistical Analysis

The data were expressed as the mean ± SD obtained for safety studies. Significance was tested using ANOVA followed by Dunnett's multiple comparison test. For metformin analysis, linear regression analysis was used to find concentration of metformin at different time points in plasma. Mean of AUC ± SEM was calculated using GraphPad Prism version 4.00 for Windows, GraphPad Software, USA. Statistical significance was accepted at *P* <  0.05.

## 3. Results

### 3.1. Phytochemical Standardization

CA extracts are standardized for polyphenols based on the relative retention and spectra match. Polyphenols such as (–) epicatechin, procyanidin B1 and (–) catechin were quantified in CA-HA extract (Supplementary Figure 1), while (–) catechin and (–) epicatechin gallate in CA-SFE extract (Supplementary Figure 2). Polyphenols quantified in CA-HA are epicatechin (14%), catechin (4.5%) and procyanidin B1 (1%), while CA-SFE contains catechin (6%) and epicatechin gallate (20%).

### 3.2. Safety Pharmacology

No mortality was observed during the treatment period of 12 weeks in either the control or treated groups. The animals did not show any changes in general behavior and physiological activities. No change was observed in the body and organ weight between control and CA-dosed animals of both sexes. No significant changes in the blood hematological values were observed (Supplementary Table 1). No significant changes in the differential counts were observed (Supplementary Table 2). Male rats dosed with 1000 mg kg^−1^ of CA-SFE demonstrate 30 and 12% increase in ALT and ALP, respectively (Supplementary Table 3). In the female group, on dosing with CA-SFE at 250 mg kg^−1^ a decrease in the AST by 34% was observed. No significant change in total bilirubin, TC, TG, glucose and creatinine were observed.

### 3.3. Cardiovascular Safety

An increase in dose of CA extracts caused a significant decrease in serum homocysteine (*P* <  0.01) (Table 1). A reduction of 10% was also seen in CK-MB in 1000 mg kg^−1^ dose of both CA-HA and CA-SFE. However, there was no significant change in troponin. There was no significant difference between both systolic and diastolic blood pressure between CA extract-dosed animals and controls (Table 2). ECG variables like heart rate (R-R interval), QT interval (diastolic dysfunction) and ventricular hypertrophy (R wave amplitude) were normal in all the groups (Table 3).

### 3.4. Pharmacokinetic Herb-Drug Interaction

CA-SFE extracts at a dose of 1000 mg kg^−1^ when co-administered with metformin decreased the AUC_M_ by 60% than the metformin alone (*P* <  0.01). At the same dose the maximum concentration (*C*_max_) of metformin was decreased by 55%. At a dose of 250 and 500 mg kg^−1^ CA-SFE decreased AUC_M_ by 33 (*P* <  0.05) and 30%, respectively (Figure 1(b)). CA-HA extracts at doses of 500 and 1000 mg kg^−1^ did not significantly change in AUC_M_ as compared with the metformin group (Figure 1(a)). There was no significant change in blood glucose when compared between metformin- and CA-treated groups.

## 4. Discussion

Ethnopharmacology and traditional knowledge-inspired approaches have been useful in drug discovery and development [32, 33]. While many traditional herbal medicines available in the market use different types of extracts, Indian FDA prohibits use of solvents except hydro-alcohol in traditional Ayurvedic formulations. The advantage of traditional knowledge in herbal drug development becomes relevant and useful only when the traditional processes are strictly adopted. Use of any modern technology or solvents to prepare purified or beneficiated extracts may be needed and desirable; however, this needs to be considered as a deviation from traditional processes. The extracts or herbal drugs prepared as per traditional processes may be accepted to be generally safe. However, technology based and/or solvent extracts need to be treated as new entities and therefore they should not be generally considered as safe. Due to new extraction technologies use of concentrates, actives and total or crude extracts are becoming popular and economical. However, whether such new technology-based extracts can or should replace the traditional extraction processes is still debatable [34]. It is advocated that detailed safety and herb-drug interaction studies are necessary before such new extracts are used in herbal formulations. Over this background, the present exploratory study was undertaken on two extracts of Ayurvedic medicinal plant. We suggest a differential development strategy for traditional and technology-based extracts [35].

We studied safety pharmacology and pharmacokinetic herb-drug interaction of CA extracts at different doses for 12 weeks. Our observations indicate that the test substances did not significantly alter general physiology, body weight, food consumption, differential count and hematology. Both CA and metformin are anti-hyperglycemic agents and therefore the blood glucose in control and experimental groups may not have significantly changed. The CA-HA was well tolerated without any adverse events in both male and female rats. There was an increase in liver enzymes, ALT and ALP, in male rats fed on highest dose of CA-SFE. This increase can be classified as level 2 of the drug-induced liver injury (DILI) (a criterion used for human drug safety studies based on the percentage increase (>25–50%) in the liver enzyme levels), which is not considered to be clinically relevant [36]. Total bilirubin, which is the actual measure of liver function, was not altered in males and females. Liver histopathological findings for focal necrosis and cellular infiltration were negative in both sexes. Taken together, the study suggests that both extracts of CA are safe.

Cardiovascular safety for newer therapeutic agents has been made mandatory by the FDA. Organ-specific safety studies for drugs for treatment or prevention of type 2 diabetes is also under consideration by the FDA. Anti-diabetic activities of CA are known in the literature [37, 38]; however, its cardiovascular effects have not yet been evaluated. The ECG examinations demonstrated normal cardiac repolarization (QT interval), normal heart rate and no signs of ventricular hypertrophy. Our results indicate the heart function to be normal and rules out any occult cardiac dysfunction.

Troponin I increase during heart failure is predictive of mortality and ventricular rhythm abnormalities. Inflammatory conditions with heart muscle involvement termed as myopericarditis and cardiomyopathy increases troponin I. Recent literature suggests troponin I as better predictive marker than CKMB [39, 40]. Thus, homocysteine, troponin I and CKMB were considered as markers for cardiovascular risk. In our study, troponin I and CKMB did not demonstrate clinically relevant changes; however, a significant decrease in homocysteine indicates a causal relation. These effects on the cardiovascular system may be attributed to polyphenols and call for detailed investigations using suitable animal models [41].

**Figure 2:**
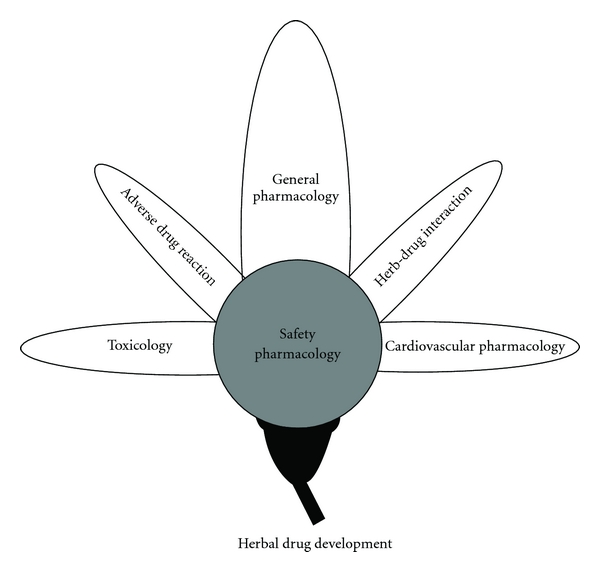
Safety pharmacology in herbal drug development.

The pharmacokinetic herb-drug interaction is an important aspect which needs be included in safety pharmacology studies (Figure 2). It is predicated that herb-drug interactions may be under-reported, under-estimated and probably may occur more frequently than the drug-drug interaction [42]. The CA-HA extract when co-administered with metformin did not interfere with pharmacokinetics. However, co-administration with CA-SFE extract at 250 (*P* <  0.05), 500 (*P* <  0.01) and 1000 mg kg^−1^ (*P* <  0.05) decreased metformin concentration, at the earliest time point of 15 min. This trend continues in CA-SFE 500 and 1000 mg kg^−1^. In CA-SFE 500 mg kg^−1^ the *C*_max_ of metformin is delayed by 1.5 h; however; in CA-SFE 1000 mg kg^−1^ there is a significant (*P* <  0.01) decrease in metformin's *C*_max_ indicating inhibition in metformin's absorption (Figure 1(b)). This clearly suggests that the CA-SFE interferes with the absorption of metformin which can be explained by the presence of non-polar components. Metformin has negligible plasma protein binding and it undergoes active renal tubular excretion by organic cation transporter (OCT2). Modulation of OCT2 would have changed the elimination pattern; however, we did not observe any such changes when co-administered with metformin. Hence, it is recommended that co-administration of CA-SFE with modern medicine should be avoided unless supporting evidence base especially on safety and pharmacokinetics is available.

Our study indicates that preclinical safety pharmacology is needed to avoid clinical adverse drug reactions. For herbal drug development, pharmacokinetic herb-drug interaction studies should form an integral part of safety studies. We suggest that safety of technology-based extracts cannot be assumed especially when it amounts to major deviation from the traditional process. Such extracts should be treated as newer entities, and basic safety and pharmacokinetic herb-drug interaction studies are desirable.

## Supplementary Data

Supplementary data are available at *eCAM* online.

## Funding

Authors thank The Lady Tata Memorial Trust, Mumbai, India, (http://www.dorabjitatatrust.org/) that provided part funding through contingency for this work; remaining funding has come through respective institutions where the work was carried out. Supercritical extracts were prepared at gratis by Vedic Supercriticals & Biotechnologies (I) Pvt. Ltd, Pune, under supervision (R. Mogre and A. S. Puranik).

## Supplementary Material

Supplementary Table 1: Hematological parameters of animals dosed with CA extracts.
Supplementary Table 2: Differential white blood count of animals dosed with CA extracts.
Supplementary Table 3: Serum biochemistry values of animals treated with different extracts of CA.
Supplementary Figure 1: Standardization of hydroalcoholic extract of *Cassia auriculata* seeds for procyanidin B1, epicatechin and catechin.
Supplementary Figure 2: Standardization of supercritical of *Cassia auriculata* seeds for epicatechin gallate and epicatechin.

## Figures and Tables

**Table 1:** Cardiovascular risk markers demonstrating cardiovascular safety of CA dosed animals.

	Control (0.5% NaCMC)	*C. auriculata* (mg kg^−1^)				
		HA		SFE		
		500	1000	250	500	1000
Male						
Homocysteine (*μ*mol L^−1^)	10.2 ± 1.52	9.5 ± 2.15	5.8 ± 1.51**	7.7 ± 1.66	7.8 ± 3.10	5.5 ± 3.09**
CKMB (IU L^−1^)	141.6 ± 26.65	143.8 ± 33.21	120.5 ± 20.0	135.7 ± 13.33	125.6 ± 31.55	120.4 ± 13.66
Troponin I (ng mL^−1^)	0.64 ± 0.03	0.67 ± 0.05	0.56 ± 0.10	0.48 ± 0.09	0.61 ± 0.08	0.55 ± 0.06
Female						
Homocysteine (*μ*mol L^−1^)	11.0 ± 2.52	10.0 ± 1.52	6.6 ± 1.74*	6.9 ± 1.35	9.2 ± 1.85	6.5 ± 2.14*
CKMB (IU L^−1^)	150.2 ± 30.27	138.9 ± 24.22	133.8 ± 15.88	145.5 ± 33.20	132.2 ± 30.9	135.5 ± 16.3
Troponin I (ng mL^−1^)	0.51 ± 0.10	0.47 ± 0.07	0.56 ± 0.09	0.55 ± 0.04	0.55 ± 0.03	0.67 ± 0.04

Data are expressed as mean ± SD, *n* = 6. *P < 0.05, **P < 0.01 significant decrease in homocysteine seen by 1000 mg kg^−1^ dose of CA-HA and CA-SFE.

**Table 2:** Ventricular pressures of CA-dosed animals.

	Control (0.5% NaCMC)	*C. auriculata* (mg kg^−1^)				
Ventricular pressures (mmHg)		HA		SFE		
		500	1000	250	500	1000
Male						
Systolic blood pressure	124.2 ± 6.2	119.4 ± 9.6	117.2 ± 4.7	121.3 ± 8.1	113.1 ± 11.2	121.9 ± 3.2
Diastolic blood pressure	83.2 ± 4.3	81.0 ± 3.4	79.5 ± 4.0	82.1 ± 4.8	79.2 ± 7.01	81.9 ± 6.4
Female						
Systolic blood pressure	119.3 ± 8.21	116.2 ± 3.9	118.7 ± 3.8	120.1 ± 11.5	116.9 ± 9.21	118.3 ± 9.8
Diastolic blood pressure	78.3 ± 6.2	77.3 ± 4.1	81.8 ± 2.9	79.9 ± 3.9	81.2 ± 4.01	77.4 ± 8.3

Data are expressed as mean ± SD, *n* = 6. No significant difference ventricular pressures seen at increasing doses of both CA-HA and CA-SFE.

**Table 3:** Electro-cardiograms (ECG) variables demonstrating cardiovascular safety of CA extracts.

	Control (0.5% NaCMC)	*C. auriculata* (mg kg^−1^)				
		HA		SFE		
		500	1000	250	500	1000
Males						
QT interval(s)	7.0 ± 1.2	7.1 ± 1.5	6.5 ± 0.6	7.0 ± 0.5	7.5 ± 0.3	6.8 ± 2.5
R wave amplitude (*μ*V)	225 ± 66	250 ± 65	200 ± 69	250 ± 55	300 ± 54	350 ± 48
R-R interval(s)	0.3 ± 0.01	0.25 ± 0.01	0.22 ± 0.05	0.29 ± 0.03	0.22 ± 0.08	0.28 ± 0.09
Females						
QT interval(s)	7.5 ± 1.9	6.0 ± 1.0	7.2 ± 0.4	7.2 ± 0.8	7.9 ± 0.9	6.0 ± 1.2
R wave amplitude (*μ*V)	255 ± 92	220 ± 55	255 ± 169	253 ± 68	210 ± 120	220 ± 96
R-R interval(s)	0.22 ± 0.02	0.19 ± 0.02	0.20 ± 0.03	0.23 ± 0.03	0.29 ± 0.05	0.30 ± 0.09

Data are expressed as mean ± SD, *n* = 6. No significant difference in heart rate (R-R interval), QT interval (diastolic dysfunction) and ventricular hypertrophy (R wave amplitude) observed at increasing doses of both CA-HA and CA-SFE demonstrates safety of CA extracts.
