# Supplementary material for: Cassia auriculata: Aspects of Safety Pharmacology and Drug Interaction
Source: Evid Based Complement Alternat Med. 2011 May 3;2011:915240. doi: 10.1093/ecam/nep237 (PMC3137796; doi:10.1093/ecam/nep237)
Supplement: Supplementary file 1 — Supplementary Table 1: Hematological parameters of animals dosed with CA extracts. Supplementary Table 2: Differential white blood count of animals dosed with CA extracts. Supplementary Table 3: Serum biochemistry values of animals treated with different extracts of CA. Supplementary Figure 1: Standardization of hydroalcoholic extract of Cassia auriculata seeds for procyanidin B1, epicatechin and catechin. Supplementary Figure 2: Standardization of supercritical of Cassia auriculata seeds for epicatechin gallate and epicatechin. [file 915240.f1.pdf]

Supplementary Table 1

Hematological parameters of animals dosed with CA extracts.

Hematological parameters	Control (0.5 % NaCMC)	*C. auriculata* (mg kg^{-1})				
		HA		SFE		
		500	1000	250	500	1000
**Male**						
WBC (X10^3 μL^{-1})	11.8 ± 3.0	10.5 ± 3.9	7.1 ± 2.4	12.3 ± 3.2	7.2 ± 0.5	15.9 ± 2.5
RBC (X10^6 μL^{-1})	8.7 ± 1.2	8.07 ± 1.0	7.7 ± 0.9	7.4 ± 1.0	7.1 ± 0.6	7.9 ± 0.2
HB (g dL^{-1})	16.3 ± 1.3	15.7 ± 1.3	14.80 ± 1.1	14.0 ± 1.0	12.7 ± 1.6	14.4 ± 0.6
HCT (%)	50.9 ± 6.3	48.7 ± 5.6	46.3 ± 4.2	44.3 ± 4.2	40.4 ± 4.4	45.3 ± 1.6
MCV (fL)	58.6 ± 2.2	60.5 ± 1.1	59.0 ± 1.3	60.7 ± 2.7	57.1 ± 1.9	57.7 ± 2.4
MCH (pg)	18.9 ± 1.4	18.8 ± 0.8	17.9 ± 0.6	19.4 ± 0.9	17.9 ± 1.3	18.3 ± 1.0
MCHC (g dL^{-1})	32.2 ± 1.5	32.3 ± 1.1	32.0 ± 0.59	31.5 ± 0.7	31.4 ± 1.3	31.7 ± 0.8
PLT (X10^3 μL^{-1})	656.8 ± 191.4	662.8 ± 142.2	673 ± 142.6	678.3 ± 233.9	671.2 ± 266.1	701.2 ± 196.3
**Female**						
WBC (X10^3 μL^{-1})	7.8 ± 3.2	6.6 ± 1.1	6.7 ± 1.7	7.2 ± 3.0	8.0 ± 2.9	8.5 ± 3.0
RBC (X10^6 μL^{-1})	7.6 ± 1.4	7.8 ± 0.8	8.2 ± 0.3	8.29 ± 0.8	8.07 ± 1.0	8.8 ± 1.4
HB (g dL^{-1})	15.6 ± 1.3	14.8 ± 1.1	14.6 ± 1.1	15.15 ± 1.80	14.1 ± 0.4	13.5 ± 1.6
HCT (%)	48.7 ± 5.08	46.3 ± 4.2	45.8 ± 3.3	48.0 ± 6.3	49.1 ± 8.1	46.2 ± 4.0
MCV (fL)	60.4 ± 1.0	59.0 ± 1.3	56.18 ± 1.7	57.8 ± 2.3	60.6 ± 5.7	62.4 ± 6.6
MCH (pg)	19.5 ± 0.9	18.90 ± 0.8	17.9 ± 0.7	18.2 ± 0.5	20.3 ± 1.0	19.9 ± 0.8
MCHC (g dL^{-1})	32.3 ± 1.0	31.9 ± 0.6	32.0 ± 0.3	31.6 ± 0.5	34.1 ± 1.1	34.0 ± 0.8
PLT (X10^3 μL^{-1})	600.8 ± 150.0	562.8 ± 130.2	620.4 ± 129.6	550.2 ± 166.1	612.2 ± 106.4	652.2 ± 95.9

Data are expressed as mean ± S.D., n = 6, No statistical difference between the control and CA extracts treated rats.

Supplementary Table 2

Differential white blood count of animals dosed with CA extracts.

Differential count (%)	Control (0.5 % NaCMC)	*C. auriculata* (mg kg^{-1})				
		HA		SFE		
		**500**	**1000**	**250**	**500**	**1000**
Male						
Neutrophil	29.2 ± 5.3	28.4 ± 11.1	30.2 ± 9.5	31.2 ± 7.4	31.6 ± 6.3	32.3 ± 5.1
Eosinophil	5.4 ± 1.0	3.2 ± 2.1	2.3 ± 1.3	5.7 ± 0.6	5.0 ± 0.9	5.5 ± 1.1
Lymphocyte	75.8 ± 12.1	72.0 ± 9.1	61.4 ± 13.5	83.5 ± 11.3	63.7 ± 10.3	84.3 ± 11.1
Monocyte	3.9 ± 1.3	4.7 ± 2.1	3.9 ± 1.5	4.0 ± 1.5	2.1 ± 1.1	4.9 ± 0.7
Female						
Neutrophil	28.1 ± 7.2	31.2 ± 7.4	26.4 ± 10.1	32.9 ± 12.4	35.5 ± 14.1	28.1 ± 11.1
Eosinophil	4.3 ± 1.1	2.1 ± 1.1	2.7 ± 2.1	3.1 ± 2.1	5.6 ± 1.1	5.0 ± 0.8
Lymphocyte	63.4 ± 10.1	80.1 ± 15.3	73.2 ± 21.2	75.3 ± 22.5	65.1 ± 15.2	60.2 ± 21.1
Monocyte	4.0 ± 1.1	3.4 ± 2.1	3.4 ± 1.1	2.5 ± 0.9	4.6 ± 1.7	2.5 ± 1.8

Data are expressed as mean ± S.D., n = 6, No statistical difference between the control and CA extracts treated rats.

Supplementary Table 3: Serum biochemistry values of animals treated with different extracts of CA

Serum biochemistry	Control (0.5 % NaCMC)	*C. auriculata* (mg kg^{-1})				
		HA		SFE		
		500	1000	250	500	1000
Male						
Fasting Glucose (mg dL^{-1})	107.0 ± 10.4	124.7 ± 16.2	119.4 ± 11.7	104.0 ± 26.2	118.3 ± 20.7	117.9 ± 14.2
Cholesterol (mg dL^{-1})	61.2 ± 11.7	53.2 ± 8.4	58.1 ± 18.6	54.4 ± 11.4	89.0 ± 28.8	60.0 ± 12.3
Triglyceride (mg dL^{-1})	83.0 ± 25.1	74.0 ± 18.2	98.2 ± 10.1	87.6 ± 26.4	83.0 ± 32.3	85.0 ± 5.8
ALT (IU L^{-1})	64.6 ± 15.6	55.5 ± 15.1	56.4 ± 6.4	71.0 ± 14.9	63.0 ± 16.8	95.0 ± 22.7**
AST (IU L^{-1})	199.0 ± 28.3	181.0 ± 22.4	167.0 ± 13.1	186.0 ± 30.6	183.0 ± 32.4	185.0 ± 13.1
Total Bilirubin (mg dL^{-1})	0.34 ± 0.06	0.41 ± 0.05	0.40 ± 0.01	0.33 ± 0.06	0.44 ± 0.07	0.5 ± 0.1
ALP (IU L^{-1})	514.0 ± 90.0	505.0 ± 84.4	555.0 ± 97.1	491.0 ± 93.6	505.0 ± 94.7	579.0 ± 74.5*
Blood Urea Nitrogen (mg dL^{-1})	55.0 ± 4.2	53.0 ± 2.2	44.0 ± 10.1	50.0 ± 3.4	39.0 ± 10.6	44 ± 3.2
Creatinine (mg dL^{-1})	0.51 ± 0.05	0.55 ± 0.02	0.44 ± 0.03	0.49 ± 0.09	0.7 ± 0.2	0.4 ± 0.03
Total Proteins (mg dL^{-1})	8.3 ± 0.4	7.8 ± 1.0	8.0 ± 0.8	7.7 ± 0.75	7.6 ± 1.0	7.3 ± 0.6
Albumin (mg dL^{-1})	3.8 ± 0.1	3.0 ± 0.2	3.7 ± 0.3	3.4 ± 0.22	3.9 ± 0.6	3.4 ± 0.6
Globulin (mg dL^{-1})	4.5 ± 0.5	4.9 ± 0.7	4.1 ± 0.4	4.3 ± 0.65	3.6 ± 1.1	3.9 ± 0.4
Female						
Fasting Glucose (mg dL^{-1})	124.5 ± 17.6	118.3 ± 20.1	117.3 ± 10.9	155.0 ± 33.5	89.2 ± 12.2	122.1 ± 22.5
Cholesterol (mg dL^{-1})	53.2 ± 8.4	58.1 ± 18.6	51.9 ± 11.3	47.9 ± 19.1	61.2 ± 14.1	59.2 ± 21.5
Triglyceride (mg dL^{-1})	90.1 ± 21.9	104.6 ± 28.3	110.8 ± 29.8	71.5 ± 45.4	75.2 ± 22.4	84.4 ± 26.2
ALT (IU L^{-1})	55.5 ± 15.1	52.3 ± 16.4	53.4 ± 14.4	34.9 ± 21.4	60.5 ± 23.4	56.4 ± 14.1
AST (IU L^{-1})	177.9 ± 29.0	161.1 ± 31.5	156.4 ± 40.8	117.3 ± 46.7**	169.8 ± 36.5	170.4 ± 44.1
Total Bilirubin (mg dL^{-1})	0.39 ± 0.1	0.27 ± 0.09	0.33 ± 0.08	0.36 ± 0.04	0.4 ± 0.1	0.43 ± 0.06
ALP (IU L^{-1})	523.2 ± 106.4	558.5 ± 102.3	498.5 ± 112.7	470.8 ± 102.1	470.5 ± 101.5	541.0 ± 51.9
Blood Urea Nitrogen (mg dL^{-1})	51.0 ± 1.4	55.1 ± 16.1	39 ± 5.7	35.7 ± 12.7	45.4 ± 10.5	48.5 ± 11.5
Creatinine (mg dL^{-1})	0.6 ± 0.0	0.55 ± 0.04	0.53 ± 0.09	0.5 ± 0.09	0.7 ± 0.05	0.6 ± 0.07
Total Proteins (mg dL^{-1})	8.1 ± 0.5	7.9 ± 0.9	7.8 ± 0.8	7.4 ± 2.8	7.7 ± 1.5	8.2 ± 1.2
Albumin (mg dL^{-1})	3.6 ± 0.2	3.5 ± 0.3	3.6 ± 0.2	3.1 ± 1.3	3.5 ± 1.1	3.6 ± 0.8
Globulin (mg dL^{-1})	4.5 ± 0.5	4.4 ± 0.7	4.1 ± 0.61	4.2 ± 1.6	4.0 ± 1.5	4.2 ± 0.8

Data are expressed as mean ± S.D., n = 6, statistical difference between the control and CA extract treated rats denoted by *$P < 0.05$, ** $P < 0.01$.
