# Supplementary material for: Cassia auriculata: Aspects of Safety Pharmacology and Drug Interaction
Source: Evid Based Complement Alternat Med. 2011 May 3;2011:915240. doi: 10.1093/ecam/nep237 (PMC3137796; doi:10.1093/ecam/nep237)
Supplement: Supplementary file 3 [file 915240.f3.pdf]

Supplementary Figure 2. Standardization of supercritical fractions of *Cassia auriculata* seeds for epicatechin gallate and epicatechin

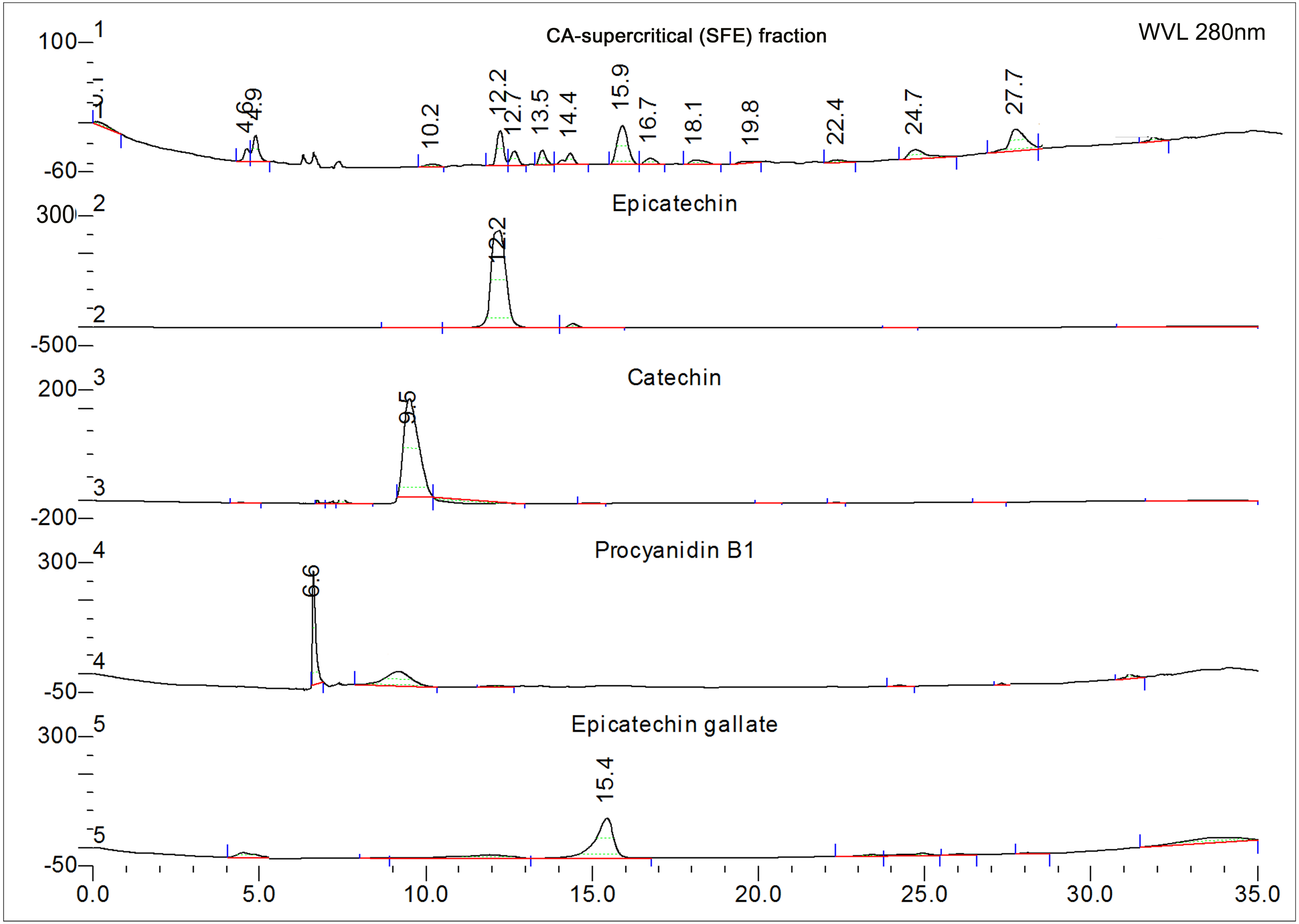

Mobile phase A – water: methanol: formic acid (79.5:20:0.5) and B- acetonitrile: formic acid (99.7:0.3) at a flow rate of 0.4 mL. Gradient was as follows: 0 to 15 min – B (5%); 15 to 20 min – B (5 to 20%); 20 to 25 min – B (20 to 30%); 25 to 30 min –B (30 to 50%); 30 to 35 min – re-equilibration B (50 to 5%)

Relative retention at 280nm and the spectra match demonstrates presence of epicatechin gallate (20%) and catechin (6%) in supercritical fraction of *Cassia auriculata* seeds
